# Extrusion Parameters Optimization and Mechanical Properties of Bio-Polyamide 11-Based Biocomposites Reinforced with Short Basalt Fibers

**DOI:** 10.3390/polym16213092

**Published:** 2024-10-31

**Authors:** Vito Gigante, Francesca Cartoni, Bianca Dal Pont, Laura Aliotta

**Affiliations:** 1 Department of Civil and Industrial Engineering, University of Pisa, Via Diotisalvi 2, 56122 Pisa, Italy; vito.gigante@unipi.it (V.G.); bianca.dalpont@phd.unipi.it (B.D.P.); 2 Interuniversity National Consortium of Materials Science and Technology (INSTM), Via Giusti 9, 50121 Florence, Italy

**Keywords:** biocomposites, basalt fibers, extrusion optimization, IFSS, analytical models

## Abstract

The increasing demand for sustainable materials in high-value applications, particularly in the automotive industry, has prompted the development of biocomposites based on renewable or recyclable matrices and natural fibers as reinforcements. In this context, this paper aimed to produce composites with improved mechanical and thermal properties (tensile, flexural, and heat deflection temperature) through an optimized process pathway using a biobased polyamide reinforced with short basalt fibers. This study emphasizes the critical impact of fiber length, matrix adhesion, and the variation in matrix properties with increasing fiber content. These factors influence the properties of short-fiber composites produced via primary processing using extrusion and shaped through injection molding. The aim of this work was to optimize extrusion conditions using a 1D simulation software to minimize excessive fiber fragmentation during the extrusion process. The predictive model’s capacity to forecast fiber degradation and the extent of additional fiber breakage during extrusion was evaluated. Furthermore, the impact of injection molding on these conditions was investigated. Moreover, a comprehensive thermomechanical characterization of the composites, comprising 10%, 20%, and 30% fiber content, was carried out, focusing on the correlation with morphology and processing using SEM and micro-CT analyses. In particular, how the extrusion process parameters adopted can influence fiber breakage and how injection molding can influence the fiber orientation were investigated, highlighting their influence in determining the final mechanical properties of short fiber composites. By optimizing the process parameters, an increment with respect to bio-PA11 in the tensile strength of 38%, stiffness of 140%, and HDT of 77% compared to the matrix were obtained.

## 1. Introduction

In recent years, the circular economy concept [1] and the production of “circular-by-design” materials [2] has sparked growing interest in producing composite materials using biobased and natural components for the different application sectors. This includes sectors where the final product is required to exhibit consistent and durable mechanical properties over time [3].

In this context, the use of short basalt fibers as reinforcing agents is gaining increasing attraction in both academia and in industry [4]. Basalt fibers, derived from natural basalt rock, are considered non-destructive and safe for the human body [5]. Initially produced by the Moscow Research Institute of Glass and Plastic in 1953–1954, large-scale production began in the 1990s, mainly in Ukraine and Australia [6]. Current research focuses on reducing the production costs and expanding commercial use, with basalt fibers priced at approximately USD 5 per kilogram compared to USD 15 per kilogram for glass fibers and USD 30 per kilogram for carbon fibers [7]. Basalt fibers are primarily composed of SiO_2_ (46–52%), Al_2_O_3_ (15–17%), Fe_2_O_3_, and FeO (9–12%) [8]; with a bulk density of 2.7 g/cm^3^ [9], basalt fibers can improve the overall performance of thermoplastic polymer matrices.

Basalt fibers, indeed, exhibit excellent resistance to high temperatures, ranging from −200 °C to 800 °C, and are durable against acid and solvent attacks [10]. Their incorporation into polymer matrices enhances sound insulation and vibration resistance [11]. A significant advantage over glass fibers is their lower moisture absorption and reduced susceptibility to shrinkage [12]. Compared to carbon fibers, basalt fibers offer cost benefits and superior flexural strength [13] and are also characterized by an easier recycling and end of life pathway [14].

Notably, studies on basalt fiber-reinforced polymers have demonstrated strong adhesion, particularly with polyamide matrices, due to the polar nature of amide groups [15]. From the point of view of the mechanical reinforcement that basalt fiber can provide to polyamide matrices, they are particularly interesting because they are comparable to the equivalent synthetic glass fibers. As demonstrated by Bednarowski et al. [12], by adding glass and basalt fibers in wt.% of 15, 30, and 50% on bio-polyamide 4.10, it was shown that basalt fibers comprise a reinforcement that provides very high strength properties, higher than that of the used glass fibers at the same level of composite filling.

There are applications, particularly in the automotive field, where the creation of components with specific geometries necessitates the use of injection molding as the preferred process [16,17]. In such cases, it is necessary to use short basalt fibers, efficiently compounded into a thermoplastic matrix.

Building on these concepts, this work aimed to develop biocomposites using chopped basalt fibers incorporated into a biobased polyamide 11 (bio-PA11). The selected matrix, chosen for its enhanced sustainability footprint, presents not only a remarkable mechanical strength, flexibility, and chemical resistance [18], but what makes it truly outstanding is its sustainable origin [19]. Bio-PA11 is produced from renewable castor oil obtained from castor bean seeds, and the remaining castor bean material is employed as a high efficiency biofertilizer. Moreover, because this material contains fewer hydrogen bonds than the more common PA6 and PA66, it exhibits lower water absorption [20]. This quality makes it more resistant to degradation from water and temperature during melt-processing conditions, which is particularly beneficial for processes like extrusion and injection molding [21]. Following the melt processing of the bio-PA11, the material is typically semicrystalline and often comprises lamellar crystals that develop into a spherulitic superstructure. It should be noted that like other linear polyamides, the maximum crystal fraction is significantly less than 50% [22]. Unlike petroleum-based polymers, the use of bio-PA11 contributes to push toward more eco-friendly material development. However, sustainability must be coupled with the technological aspects of the materials in the case of short-fiber composites, where the achievement of properties comparable to fossil-based ones is not guaranteed [23]. For this reason, the novelty of the present work lies in the intention to establish an optimized processing pathway that ensures the compounding achieves both a dispersive and distributive mixing of basalt fibers in the bio-PA11, limiting the fiber breakage. The optimization of the extrusion compounding is of paramount importance in limiting the fiber breakage that has a significant impact on the improvement of the composite strength. The careful extrusion optimization during compounding makes possible the obtainment of a biocomposite with high stiffness (thanks to the high elastic modulus of the basalt fibers, which is around 85–87 GPa [24]) and better breaking strength thanks to the reduced fiber fragmentation [24].

A comprehensive review of the literature revealed a dearth of studies investigating the production of composites with bio-PA11 and short basalt fibers. Many studies have used nylon 6 or 6,6 as the matrix [25,26] or employed basalt fabrics as reinforcements [27,28]; Barczewski et al. [29] produced short-fiber basalt composites with PA11 focusing primarily on the recyclability advantages of these composites and how the incorporation of flame retardants into the melt could degrade the matrix itself during processing. Additionally, these studies did not consider how the initial length of short fibers, typically ranging from 3 to 12 mm [30], does not remain unchanged after extrusion and molding processes. The processability and mechanical properties of the material extrusion samples are influenced by a number of factors including fiber orientation, fiber fraction, and aspect ratio [31,32]. A significant issue, particularly in the compounding extruder and the feed zone of the injection molding screw, is the intense shearing stresses that reduce the length of glass fibers to just a few tenths of a millimeter, regardless of their original size [33]. It is therefore essential to control fiber breakage, since increasing the residual fiber length in composite products can significantly enhance the mechanical and physical properties of injection-molded items [34].

The goal of the paper was to find a compromise between fiber homogenization in the melt, preventing matrix degradation during extrusion. Additionally, it is crucial to ensure that the morphological structure remains unaltered by secondary processing (injection molding) while verifying the reliability of the extrusion process predictive software and optimizing the boundary conditions. As mentioned, during compounding, fragmentation occurs, and further breakage typically happens in the nozzle and mold during the shaping process. This frequently leads to a reduction in the average length to a range of 0.2 to 0.4 mm [35]. This length is frequently below the critical fiber length, which means that the potential benefits of fiber reinforcement cannot be fully realized by short-fiber reinforcement [36]. Indeed, to predict the fiber breakage, but also to develop and provide data on the evolution of many outputs (temperature, viscosity, shear rate) along the screw axis [37], simulation of the extrusion process through 1D software, Ludovic^®^ (version 7.2) was carried out. By setting all the geometric parameters of the screws, heat transfer coefficients, temperature of the barrels, thermal and rheological features, the software can predict the optimal primary process operating conditions [38]. Moreover, thanks to a design of experiment (DOE) approach, a complete investigation of how the variable influences the extrusion process could be undertaken [39], and in the present study, it was widely used to predict the fiber breakage to choose the operative conditions that fulfil the desired macroscopic properties. The simulation results were compared with real extrusions in a constrained range of process conditions to verify the consistency of the boundary conditions. Injection molding was then selected as the shaping method to produce bio-PA11-based biocomposites with 10, 15, and 20 wt.% of basalt fibers, checking whether the final length of the fibers had been influenced by the secondary process. On these specimens, morphological, rheological, mechanical, and thermal tests were performed to accomplish a well-rounded characterization. To better understand the mechanical results obtained, especially the tensile strength, the fiber–matrix interfacial adhesion was evaluated adopting the modified Kelly–Tyson equation [40] in which the stress at the fiber ends in short-fiber composites are not neglected, thus allowing for a better estimation of the interfacial shear stress (IFSS). The latter parameter is commonly used as an indirect measurement of the level of adhesion between the fiber and polymer [41]. The innovation of this research lies in the importance of correlating primary and secondary process parameters with the mechanical and thermal properties of short basalt fiber composites, focusing on the fragmentation of basalt fibers caused by the extrusion compounding process. By using Ludovic^®^, fiber breakage was limited, achieving bioPA11-based composites with improved mechanical and thermal performance, which can broaden the application range of these materials.

## 2. Materials and Methods

### 2.1. Materials

The biobased polyamide 11 (bio-PA11) matrix was supplied by NaturePlast (Ifs, France) with the trade name NP BioPA11-254 [MVR (235 °C, 2.16 kg) = 30 cm^3^/10 min, density = 1.03 g/cm^3^). It is a thermoplastic resin, with a biobased content of 97% (according to the ASTM D6866 [42]) and is specifically designed for injection applications. It is well-known that polyamide is hygroscopic since water molecules can coordinate around polar amide groups [43], which can reduce its mechanical and thermal properties; for this reason, drying conditions are necessary for proper storage to avoid water absorption. In this work, the pellets were dried for six hours at 90 °C in a DPM 604 dryer supplied by Piovan Group (Venezia, Italy).

Short basalt fibers were supplied by Stewabas (465/6, STW, Kautzmann, Germany) with a nominal length of 6 mm and an average elastic modulus of 80 GPa. These fibers were produced by heating molten basalt to 1400 °C and by spinning it into filaments. These filaments usually have a diameter in the range 10–20 μm and are then coated with a silane sizing agent. Finally, the filaments are spun into yarn and subsequently chopped into short fibers.

The biocomposites produced had the compositions shown in Table 1.

### 2.2. Primary and Secondary Processing

**Figure 1:**
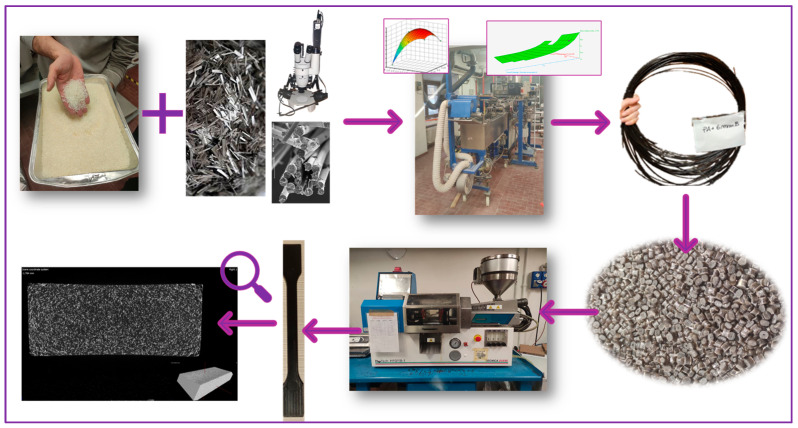
Overview of the entire manufacturing process of the short basalt fiber-based composites.

The optimized extrusion conditions and method to achieve them will be discussed in Section 3.1. This section outlines the procedure undertaken to adjust the extrusion conditions to minimize fiber breakage and enhance the quality of biocomposites produced with 10%, 15%, and 20% wt.% of basalt fibers. This procedure comprised an initial phase of process simulation and design of experiments (DoE) conducted with Ludovic^®^ (SC-Consultants, Saint Etienne, France) with the objective of delivering a comprehensive thermo-mechanical analysis of compounding within a twin-screw extruder. Once the parameters were optimized, the production of composite pellets was carried out using a semi-industrial COMAC EBC 25HT twin-screw extruder (Milano, Italy), equipped with two co-rotating screws in 11 barrels with an L/D ratio of 44 (D = 25 mm), a centerline distance of 21 mm, and a leakage of 0.1. The values were also employed in the simulation process, also taking the screw elements into account. The initial part includes conveying elements with progressively decreasing pitch, which facilitates transport of the solid material into the melting zone. Subsequently, kneading elements with different orientations are introduced to facilitate and accelerate the melting process. This is followed by a series of alternating mixing and conveying elements to ensure both distributive and dispersive mixing throughout the extrusion process until the end. At the outlet, two conveying elements with a tighter pitch are used to increase the outlet pressure. The specifics related to the precise screw elements used in the geometry profile have been indicated in a previous work [39]. Bio-PA11 was introduced into the starting point of the extruder, whereas the subsequent addition of basalt fibers was carried out through a volumetric side feeder after the first melting elements. Venting was ensured by a stripping section with a vacuum pump in the second-to-last barrel. The extruded strands were cooled in a water bath maintained at room temperature and then cut into pellets using an automatic knife cutter. Pellets obtained were finally dried for six hours at 90 °C in the above-mentioned PIOVAN dryer to prevent moisture absorption, detrimental to the mechanical properties of polyamide. Subsequently, they were employed to carry out injection molding using a Megatech H10/18-1 (Tecnica Duebi S.r.l, Fabriano, Italy) injection molding machine. As suggested by the technical datasheet of PA11, the mold temperature was set at 25 °C. The samples were all shaped according to ISO 527-1A [44] according to the injection molding parameters reported in Table 2; before testing, all samples were placed in a controlled atmosphere chamber for two days at T = 25 °C and relative humidity of 50%. A scheme of the entire manufacturing process is represented in Figure 1.

### 2.3. Optical and SEM Analysis

Basalt fibers were measured with an optical microscope (Leica DVM6, Wetzlar, Germany) to obtain length distributions (at least 300 fibers were measured) used as input for the simulations that will be discussed in Section 3.1. The fibers were carefully handled with metal tweezers, one by one, to avoid breakage, and placed on a glass slide under the microscope to measure their lengths. The fiber diameter, was instead evaluated with a scanning electron microscope (SEM TM 3000 Hitachi, Tokyo, Japan), operating under high vacuum conditions on metallized samples. Subsequently, the images were analyzed with ImageJ^®^ (version 1.54k), and the initial fiber length and diameter distributions were obtained (necessary as input for Ludovic^®^).

Indeed, post-extruded biocomposite pellets were placed in a ceramic crucible and subjected to 600 °C for 15 min in a muffle furnace (Nabertherm LHT 02/17, Lilienthal, Germany) to achieve complete burning of the polyamide matrix without damaging the fibers as suggested by Asodeh et al. [45]. To avoid abrupt temperature changes, a heating/cooling cycle was implemented before and after the isothermal treatment at 600 °C. After this pyrolysis process, the basalt fibers formed an entangled mass, which was carefully separated manually on a paper sheet and then attached to sample holders for SEM analysis.

Moreover, to better detect and observe adhesion between the bio-PA11 matrix and basalt fibers, SEM analysis was also carried out on the injection-molded specimens; the samples were immersed in liquid nitrogen and then fractured to create a smooth fracture surface.

### 2.4. Imaging—Microtomography (μ-CT)

X-ray computed tomography was carried out to investigate the internal microstructure of the injection-molded biobased polyamide 11/basalt fiber composites manufactured in this work. The samples were parallelepiped-shaped, cut from dog bone specimens, with dimensions of 80 mm × 10 mm × 4 mm. X-ray micro-CT measurements were performed using a Phoenix V|tome|x X-ray microscanner (Waygate Technologies, Baker Hughes Company, Houston, TX, USA) . The system consists of a microfocus sealed X-ray tube operating at 300 kV/500 W, equipped with a detector featuring a 200 μm pixel resolution.

### 2.5. Mechanical Characterization

Tensile properties of composite samples were evaluated according to ASTM D3039 [46] using an MTS Criterion model 43 (MTS Systems Corporation, Eden Prairie, MN, USA) equipped with a 10 kN load cell and interfaced with a computer running MTS Elite Software (version 4.0). The crosshead speed was set to 2 mm/min for evaluating the Young’s modulus up to 0.8 mm of elongation, utilizing an extensometer for more accurate measurement. Once elongation exceeded this value, the extensometer was removed, and the test continued at a rate of 10 mm/min.

Flexural properties, according to ASTM D790 [47], were assessed via a three-point bending test on the same MTS machine with a 10 kN load cell, and the crosshead speed was set to 2 mm/min. A support span of 64 mm was used, and rectangular specimens measuring 80 × 10 × 4 mm were employed.

Charpy impact tests were performed on an Instron CEAST 9050 machine (INSTRON, Canton, MA, USA) equipped with a 15 J Charpy pendulum and DAS 8000 Junior for data acquisition. The specimens measured 80 mm × 10 mm × 4 mm, obtained by cutting dog bone injection-molded samples, and were V-notched in the middle using a V-notch manual cutter type A, according to ISO 179 [48] (45° notch, 2 mm depth, and 0.25 mm radius of curvature at the base of the notch).

Finally, HDT tests were conducted using an HDT testing machine (HVT302B, MP, Milan, Italy). Parallelepipedal shaped samples were obtained by cutting dog bone specimens with dimensions of 80 mm × 10 mm × 4 mm, then positioned in a three-point bending configuration inside an oil bath with increasing temperature. The constant flexural stress employed was 0.45 MPa at the midpoint of the flatwise position, and a temperature increase of 120 °C/h was applied according to ISO 75, type B. The test was considered complete when the deflection at the midpoint reached 0.34 mm.

For all of the mechanical tests, at least five samples were tested for each composition and the average values reported.

### 2.6. IFSS Evaluation: Theoretical Background

The quality of the adhesion between fiber and matrix mostly affects the efficiency of load transfer from the matrix to the fiber, which is one of the most important parameters for obtaining matrix reinforcement [49]. The description of the interfacial properties is usually ascribed to the interfacial shear strength (IFSS), indicated along the text with τ. However, the experimental techniques used to evaluate the interfacial shear strength are not straightforward to perform correctly and consistently when working with short fibers, especially with waste derived or natural fibers [50]; for this reason, it is necessary to develop analytical or numerical models to predict the interface behavior of short-fiber composites. In the existing literature, there are several models that describe and extrapolate IFSS, however, most of these have neglected the contribution of the stress at the fiber edges [51]. This appears to be particularly true for long continuous fibers, which offer a large available surface along their lengths, making the contribution of the stress transferred at their edges not relevant. However, for short-fiber composites, this concept becomes untrue. For this reason, Aliotta et al. [40] proposed a novel equation to calculate the interfacial shear strength, starting from the Kelly–Tyson model, modifying the boundary condition related to the hypothesis of null stress transferred in correspondence to the fiber ends [52]. The model was updated assuming that the extremities of the fiber give a constant stress contribution, called *σ*_0_, equal to the matrix yield; a modified equation of the critical fiber length (*L_c_*) was obtained (Equation (1)) [40]:

(1) $$L_{c}=\frac{\left(\sigma_{f,max}-\sigma_{0}\right)D}{2\tau }$$

where *σ_f,max_* is the maximum stress that the short fiber can bear, *D* is the fiber diameter, and *τ* is the interfacial shear stress. When short fibers are used, generally, their length is below the critical length, consequently, a new definition of the critical length was introduced in the Kelly–Tyson equation [53], which allows for the prediction of the composite stress in the case of fibers having a length below the critical length. Applying this substitution, an expression of IFSS prediction was found (Equation (2)) [40]:

(2) $$\tau =\frac{1}{a_{r}}\left[\frac{\sigma_{c}-\sigma_{m}^{\prime }\left(1-V_{f}\right)}{\eta_{0}V_{f}}-\sigma_{0}\right]$$

where *a_r_* is the fibers’ aspect ratio, *σ_c_* is the tensile strength of the composite, *V_f_* is the volume fraction of the fibers, *η*_0_ is the fiber orientation (considered equal to 3/8), and $\sigma_{m}^{\prime }$ is the stress undertaken by the matrix at the fibers’ elongation at breaking.

## 3. Results

### 3.1. Twin-Screw Extrusion Optimization and DOE Results

To guarantee consistency regarding the thermomechanical flow parameters in the local 1D computations, the screw geometry, thermal, and rheological properties of the bio-PA11 and basalt fiber features were inserted as input into Ludovic software.

**Figure 2:**
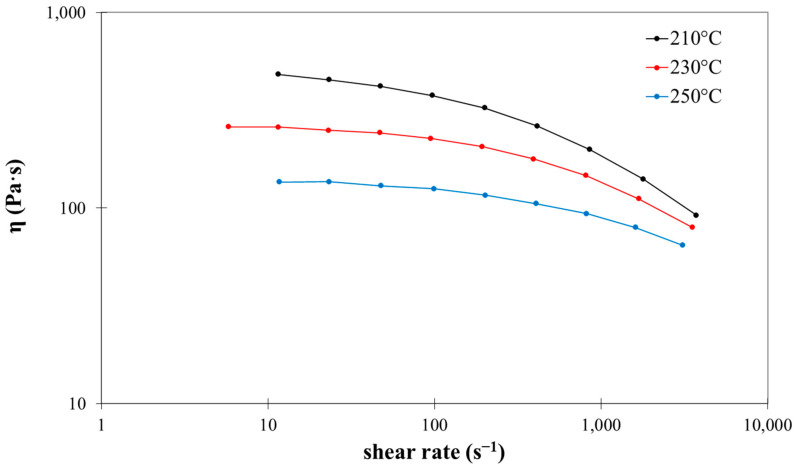
Rheological curves at three different temperatures for bio-PA11.

The viscosity of bio-PA11 was obtained from rheological measurements with a capillary rheometer (CEAST model RHEOLOGIC 2500 equipped with tungsten carbide round hole capillaries with different geometries: L/D = 10/1, 20/1, and 30/1) following the method described in [54]. In Figure 2, it is possible to observe three different curves in the function of shear rate. The black curve represents the data at T = 210 °C, the red one at T = 230 °C, and the blue one at T = 250 °C; these demonstrate the influence of temperature upon viscosity. These curves are essential for understanding the evolution of melt behavior along the extruder.

Regarding the thermal properties to be used as thermal inputs for the model, the thermal conductivities for both bio-PA11 and basalt fibers have been found in the literature [55,56]. The melting temperature and melting enthalpy were evaluated from not processed bio-PA11 granules by DSC analysis (Q200 TA instruments, Newcastle, UK), while the heat capacity was evaluated through MDSC (modulated differential scanning calorimetry) using the same instrument. All of the data are shown in Table 3. Moreover, to simulate the effect of heat exchange of the die, cylinder, and screw, a value of 1000 W/m^2^·K was defined following recommendations in the literature [57].

**Figure 3:**
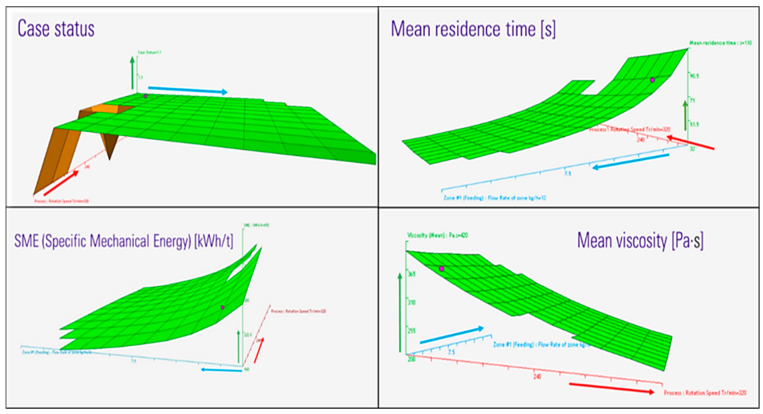
DoE results (*z*-axis in green) for the switching screw speed (red *y*-axis) and total flow rate (cyano *x*-axis).

In selecting the extrusion temperature profile, it is crucial to balance low viscosity, prevent degradation, and mixing efficiency [58]. Initially, the polymer should not be fully melted, allowing it to be conveyed to the central zone where mixing occurs. In this zone, the polymer must be completely melted to ensure optimal mixing between the biobased matrix and basalt fibers. The screw elements in this area increase the residence time of the material, thereby facilitating thorough mixing due to the low viscosity of the polymer. Furthermore, the temperature near the die is set lower than in previous zones, with the objective of increasing the melt strength. Consequently, the temperature profile selected for the extrusion simulations across the 11 barrels was as follows: 150, 170, 190, 200, 210, 220, 220, 210, 210, 200, 200, and 200 °C. To investigate the effects of the above-mentioned temperature profile, a wide-ranging design of experiments (DoE) was executed. The DoE analysis was conducted under the most challenging conditions, namely with 20% basalt fiber content. The optimized parameters identified were subsequently applied to the production of biocomposites containing 10% and 15% by weight basalt fiber. The variables selected for variation within a specified range were screw speed and total flow rate, which were divided into 11 incremental steps (respectively from 160 to 320 rpm and from 3 to 12 kg/h). Regarding the feed rate, primary parameters are those that vary independently, while secondary parameters are dependent on the primary ones. This implies that the bio-PA11 and basalt fiber flow rates represent the primary values, while the dependent values include extractions due to venting and stripping. In terms of the results, the most significant response surfaces are shown in Figure 3, where for each convergence surface, the blue axis indicates the total flow rate, the red axis indicates the screw speed while the z-axis represents the selected output. It was first ensured that all simulations converged, avoiding potential motor blockage due to excessive material processing or, conversely, insufficient material flow [59]. In this context, it is possible to notice that the case status was found to be satisfactory for all conditions except those with very low flow rates.

The DoE was subjected to an evaluation and balancing process, with a view to identify and quantify the key processing outputs. The main objectives were to minimize fiber breakage to prevent PA11 degradation. This was achieved by maintaining the lowest possible residence time and maximum temperature while also considering the total time that the melt remained at T > 230 °C. Additionally, the goal was to obtain an exit viscosity that ensured sufficient melt strength for strand formation (around 600 mPa·s) [60] to minimize the specific mechanical energy (SME) consumption, achieving a high global mixing index. In general, a low screw speed combined with high throughput has been demonstrated to be an effective approach from an energy-saving perspective, as indicated by the lower values of SME achieved. However, the optimal exit viscosity of the extruder, which is primarily dependent on screw speed, is achieved at around 200 rpm. This speed ensures adequate melt strength for proper cooling and granulation. The conditions required to prevent polymer degradation (namely, maintaining low residence times and avoiding excessively high temperatures) were achieved with low screw speed and high throughput as in other previous works [38,39]. It is important to note that RTD increases when the flow rate decreases: insufficient material in the screws can result in prolongated residence times, which may potentially lead to polymer degradation.

In Table 4, the most promising simulations were selected and the highlighted ones were used in the real process. To identify the best option for the real process, simulations with RTD < 40 s were chosen to prevent eventual bio-PA11 degradation. Additionally, this criterion ensured that the basalt fibers remained in the extruder only as long as necessary for mixing with the matrix, thereby minimizing excessive fiber breakage. A second constraint was applied, whereby only those processes where the temperature exceeded 230 °C for less than 20 s were considered.

### 3.2. Fiber Breakage Evolution Along the Extrusion Process and Comparison Predicted/Real Fiber Breakage

**Figure 4:**
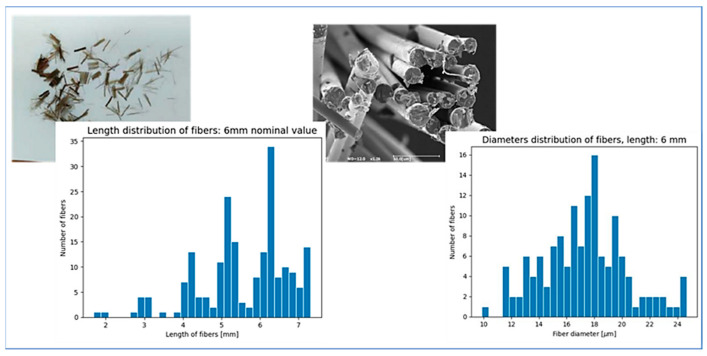
Length and diameter distributions of the basalt fibers before the compounding process.

As previously mentioned, a critical aspect of this process simulation was the analysis of fiber breakage. Ludovic^®^ software, given the elastic modulus and the initial distribution of fiber lengths and diameters, can predict the fiber breakage based on the screw geometry elements. One objective of this study was to assess the accuracy of these predictions compared to the experimental data. With the optimized parameters evaluated in Section 3.1 the predictive model for fiber breakage was computed. As shown in Figure 4, the actual distributions of fiber lengths and diameters were markedly broad and not symmetric around the mean, with an average length of 5.81 mm, an average diameter of 17.79 μm, and an average aspect ratio around 300.

To facilitate the feeding of these fibers, the side feeder included an agitator that maintained their rotational movements, ensuring a consistent supply to maintain the desired flow rate. The feeder was equipped with two screw steps that helped to convey the fibers into the extruder before mixing with the matrix. It is important to note that the feeding process itself can cause fiber breakage, which must be considered. For this reason, to gain insight into the behavior of the fibers as they are introduced into the machine, samples of fibers were taken from two different points in the extruder: immediately after the feeder and at a second extraction point. This allowed us to monitor the evolution of the fibers along the extruder including assessing how much breakage occurred before actual mixing. In this section, a comparison between simulation and real breakage was carried out; the most aggressive compounding condition was also considered, which was the extrusion with 20 wt.% of basalt fibers. The fiber distribution used as the starting point for comparison with the simulator was taken immediately after the fibers entered the extruder.

**Figure 5:**
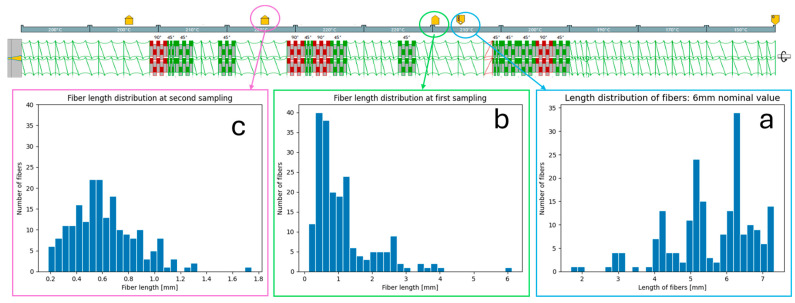
Screw geometry and (**a**) basalt fiber distribution length before extrusion; (**b**) after feeding, and (**c**) after the first mixing elements.

Figure 5 illustrates the screw geometry profile (with yellow arrows that represent, from right to left: bio-Pa11 introduction, basalt fibers feeding, two extraction points, venting through vacuum pump); at the extraction points, the material, after pyrolysis and microscopic evaluation, was used for the assessment of real fiber breakage during compounding and comparison with the Ludovic^®^ predictions.

**Figure 6:**
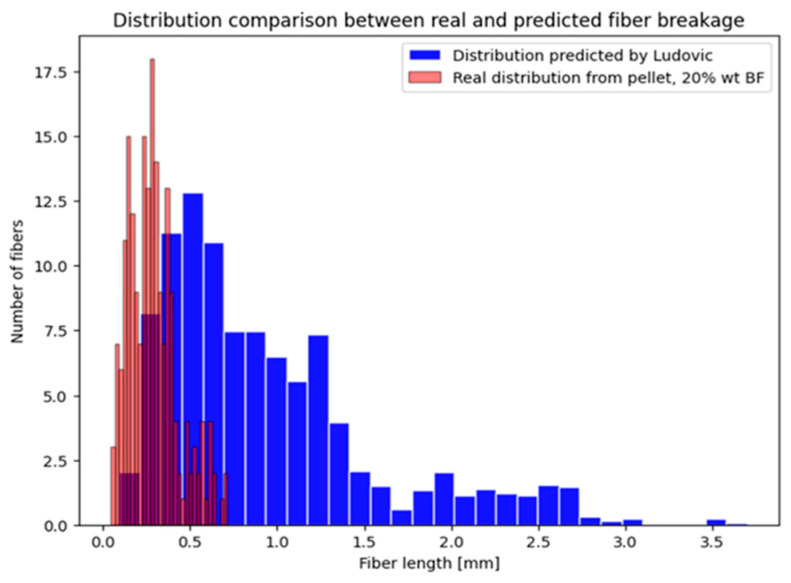
Comparison of the distribution length evaluated through pyrolysis and optical measurement (in red) and the distribution predicted by the simulation (in blue).

The results demonstrated that a notable reduction fiber length occurred within the feeder itself. Even the act of merely passing through the feeder caused a decrease in the average length, which was observed to be 1.2 mm. Unfortunately, electrostatic attraction between fibers increases their tendency to clump together, further exacerbating breakage [61]. Moreover, passing through the mixing and kneading element (three barrels) in about 15 s of residence time resulted in a decrement of fiber length to 0.6 mm, even when all the conditions were optimized through the DoE described in Section 3.1. Finally, Figure 6 provides a comparison of the final fiber distributions, post-die, both real and simulated. The Ludovic simulation was more optimistic regarding fiber breakage, yielding a distribution that differed from the actual results. While the peak could be considered similar with a slight shift on the right (avg. real length of 0.4 mm, 0.5 mm instead for the simulator), a huge difference was present in the consideration of the “tail”. The simulator suggests a higher prevalence of longer fibers, which was not observed in the real data.

**Figure 7:**
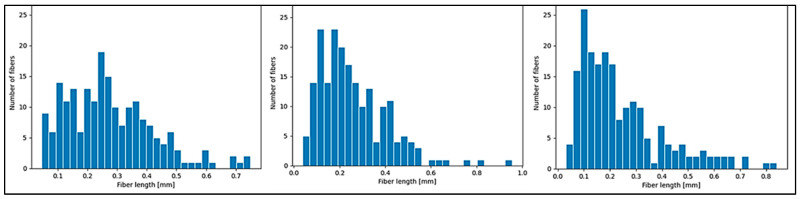
Comparison among the distribution length of injection-molded samples of biocomposites with 10, 15, and 20 wt.% of basalt fibers.

It is of fundamental interest to determine whether the process of fiber breakage is exacerbated during the injection molding process. As illustrated in Figure 7, there was a slight decrease in the average fiber length with an increase in the basalt fiber content, confirming that the production of biocomposites with the highest basalt content represents the most challenging condition. The average fiber length decreased from 0.37 mm in the biocomposite with 10 wt.% of BF to 0.34 mm in the one with 20 wt.% of BF. However, it is noteworthy that the “tail narrows”, meaning that a lower fiber content corresponds to a greater presence of a small number of longer fibers. This indicates that as the fiber content increases, there is a greater tendency for fibers to agglomerate, and due to their rigidity, they are more likely to come into contact and break [62]. Considering an unchanged value of diameters for all biocomposites after injection molding of around 16 μm, these fibers appeared to have a final aspect ratio of about 22.

### 3.3. Morphological Results

**Figure 8:**
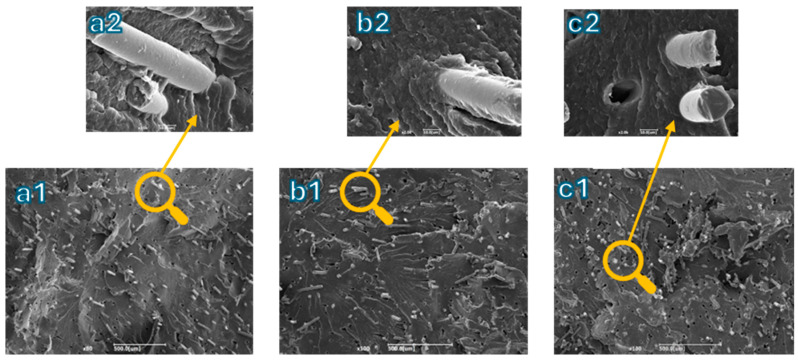
Micrograph at 80× for (**a1**) bio-PA11 + 10BF, (**b1**) bio-PA11 + 15BF, and (**c1**) bio-PA11 + 20BF. Micrograph at 2000× for (**a2**) bio-PA11 + 10BF, (**b2**) bio-PA11 + 15BF, and (**c2**) bio-PA11 + 20BF.

In Figure 8, two different magnifications are presented for each produced biocomposite. Investigation of the morphology included considering how the fibers were dispersed and distributed in the final product to connect this aspect with the mechanical properties.

Indeed, the strength of a fiber-reinforced composite in the fiber direction is influenced by the adhesion and the interfacial behavior between the fiber and the matrix [63].

Although both debonding and pull-out were observed, with the latter evidenced by the presence of holes, the dispersion of fibers within the matrix appeared to be very good. The fibers were well-homogenized without agglomerations, confirming that the optimization of the global mixing index achieved with Ludovic^®^ was maintained during the injection molding process. It appears that the fiber/matrix adhesion was pretty good, as the fibers were partially covered with the polymer, indicating a decent level of adhesion [64]. Indeed, the polymer coverage helped to fill the surface irregularities of the fibers, improving mechanical adhesion by helping to evenly distribute stresses throughout the composite. Without adequate coverage, these irregularities are not filled, reducing adhesion [65]. When the fiber content increased, the total surface area of the fibers exposed to the polymer matrix grew, which consequently caused a decrease in tensile strength with the composite with 15 wt.% of basalt.

**Figure 9:**
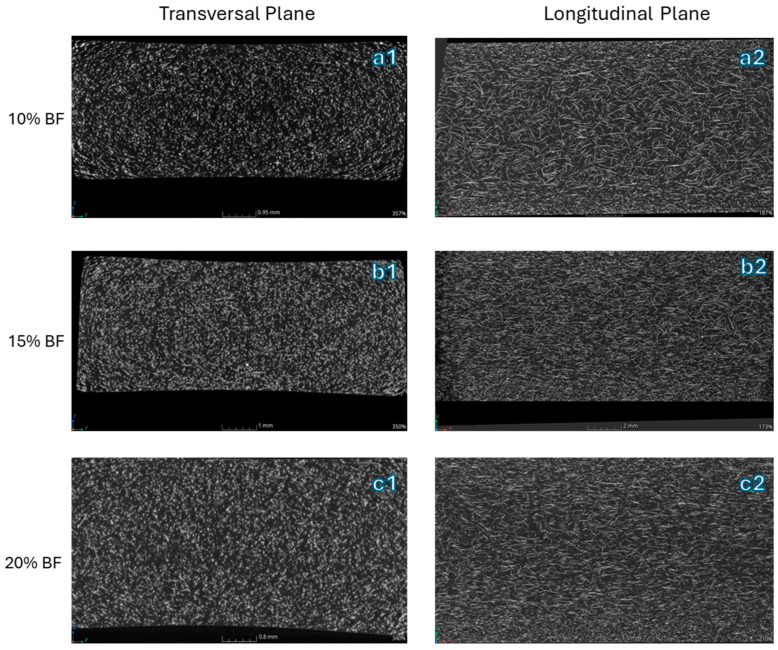
Micro-CT images showing transversal sections of (**a1**) bio-PA11 + 10BF, (**b1**) bio-PA11 + 15BF, and (**c1**) bio-PA11 + 20BF and longitudinal sections of (**a2**) bio-PA11 + 10BF, (**b2**) bio-PA11 + 15BF, and (**c2**) bio-PA11 + 20BF.

Figure 9 illustrates the micro-CT images of the biocomposites, presented from three different perspectives. In these images, the fibers are rendered white, while the matrix is black, thanks to the contrast provided by the reconstruction parameters. The injection molding process revealed several distinct layers, each exhibiting different fiber alignments. In the skin layer, fibers were typically strongly oriented in the direction of the melt flow due to the elongational forces at the melt front and the shear flow after the front has passed. In contrast, a random in-plane fiber alignment was observed in the core layer, attributed to the slower cooling rate and lower shear forces [66].

In the biocomposites under consideration, the fibers were randomly oriented in the skin layers but aligned with the flow direction in the core layer. This suggests that the fibers were subjected to high shear stresses in the direction of the flow during the injection molding process, indicating sufficiently homogeneous fiber dispersion. In the core section, a notable transition was observed from the skin to the core layer, with no significant differences observed between the morphologies of the composites containing 10 wt.% and 15 wt.% basalt fibers. Furthermore, the fiber alignment was found to be similar.

This consistency is reflected in the similar mechanical properties obtained, as will be shown in the Section 3.5. However, a notable difference appeared in the 20 wt.% of basalt fiber; in these samples, there was no clear distinction between the core and skin layers in terms of fiber orientation. This could be due to increased steric hindrance as the fiber content rises, preventing the fibers from aligning properly in the flow direction.

### 3.4. IFSS Estimation

After optical evaluation of the SEM micrographs, the adhesion was found to be good, although it needs to be improved. As discussed in the theoretical background of Section 2.6, the quality of adhesion between the fiber and matrix significantly affects the efficiency of load transfer from the matrix. Consequently, the evaluation of interfacial shear strength (IFSS) is of particularly importance. In this study, the modified Kelly–Tyson equation [40] was employed, obtaining an IFSS value of 14.2 MPa. The obtained value was consistent with the optical evaluation, as many fibers exhibited optimal adhesion, while some showed cavities around the fiber/matrix interface. Furthermore, for a polypropylene (PP) matrix reinforced with short basalt fibers, which had a similar elastic modulus to the one in the present paper, reported an IFSS value comparable to the one obtained in this study [67]. The quality of adhesion is of peculiar importance, particularly for fibers shorter than the critical length. In the present work, it was also demonstrated that the exclusion of the contribution of the ends of short fibers was no longer justifiable. Indeed, calculating the IFSS using the classical Bader and Bowyer method [68], which disregards the contribution of fiber ends [69], yielded a value of approximately 5 MPa, a result incompatible with both the morphological observations and literature data.

### 3.5. Results of Mechanical Properties

Mechanical tests were conducted to assess the properties of the biocomposites and to understand how the incorporation of basalt fibers, through an optimized processing pathway, modified the bio-PA11 characteristics.

**Figure 10:**
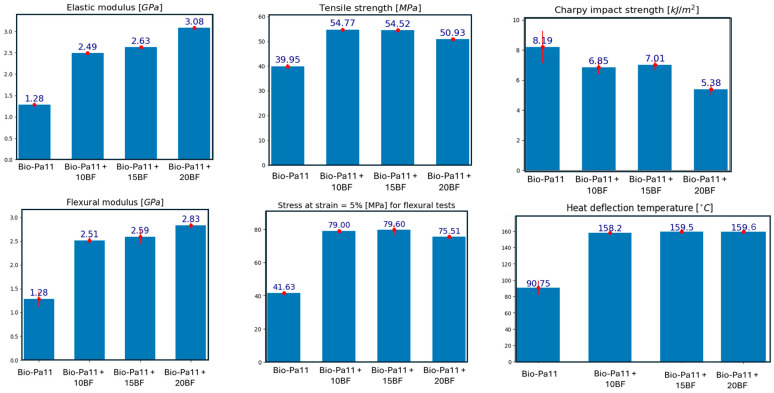
Mechanical properties of the biocomposites produced.

**Figure 11:**
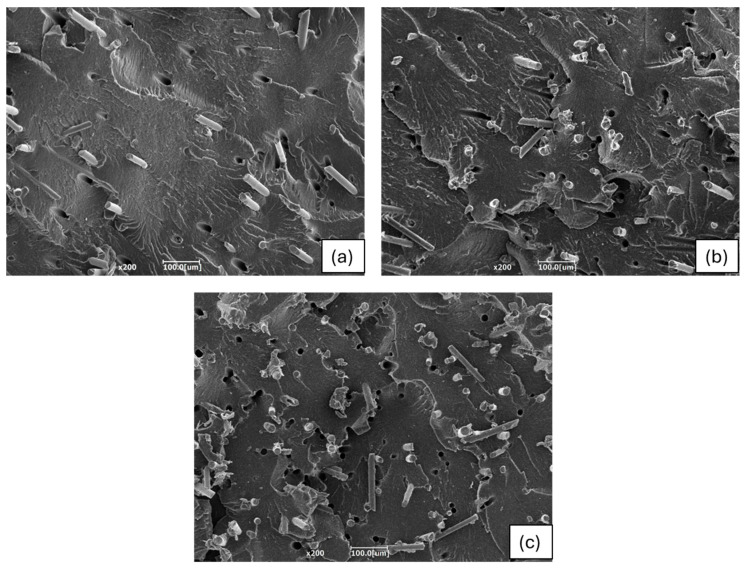
SEM magnification at 200x of the cryofractured surfaces of (**a**) bio-PA11 + 10BF, (**b**) bio-PA11 + 15BF, and (**c**) bio-PA11 + 20BF.

Figure 10 depicts the tensile, flexural, impact, and HDT results of the injection-molded samples in relation to the amount of basalt fibers. First, it was possible to notice that the elastic modulus increased with a rising fiber content. While this property was enhanced, a reduction in elongation at break was also observed. As described in [70], the addition of basalt fibers with a high elastic modulus is highly effective in improving the tensile modulus of composites, whether the matrix is nylon or polypropylene. Basalt fibers, similar to glass fibers, are particularly compatible with a polyamide matrix due to their polar nature, which enhances adhesion [71]. Considering that the stiffness of bio-PA11 is around 1.3 GPa, the incorporation of the 20 wt.% of short basalt fibers enabled an increase in the elastic modulus until 3 GPa, with an elongation at break acceptable for this kind of biocomposite (around 6%). Regarding the tensile strength, a similar trend was also observed; specifically, the values were consistent for fiber contents of 10% and 15% by weight. However, for the composite with 20wt.% basalt fiber content, the tensile strength was slightly lower. This decrease was generally attributed to the fact that excessive fiber content can reduce fiber–matrix adhesion, leading to diminished strength, as demonstrated in the literature [72,73] and as evidenced by the optical analysis. It can be seen from the SEM micrographs reported in Figure 11 how increasing the fiber content increases the number of fibers for which debonding occurs, as evidenced by the increased number of holes per unit area with the increase in basalt content.

Regarding the flexural properties, the flexural modulus followed the trend of the tensile tests; indeed, it was higher in biocomposites with greater fiber content. This was due to the improved load transfer from the matrix to the reinforcement as the fiber content increased, as highlighted by Khandelwal et al. [11]. Since the samples did not break during the test, it is inappropriate to discuss flexural strength. It was observed that the samples did not break under maximum load, likely due to the basalt fibers acting as a “bridge” during crack propagation under loading conditions. The reinforced basalt fiber composite could bear higher loads and support multiple cracks throughout the loading process. Nevertheless, to analyze the results, the histogram presented the stress values at 5% strain, as suggested by the ASTM D-790 standard test methods for the flexural properties of unreinforced and reinforced plastics. The data indicate that the addition of basalt fibers significantly improved not only the flexural modulus, but also the composite’s resistance up to 5% strain. In the analysis by Song et al. [74], the increase in flexural strength of a nylon matrix reinforced with basalt fibers was attributed to the combined effect of the high flexural strength of the nylon, which coupled with the good load-bearing capacity of the basal fibers, withstood failure due to shear and helped to distribute the applied load across the composite. Moreover, the increment in the flexural strength can also be ascribed to the good interfacial adhesion that, as reported Li et al. [75], also plays a crucial role in enhancing the flexural strength of the final composite.

The increase in flexural modulus is closely linked to the HDT increment observed in Figure 10. This behavior has already been found in the literature [34,76] and is ascribed to the fact that HDT is a three-point bending test crucial for composite design; it indicates the upper limit of dimensional stability and material strength under a fixed load across a specific temperature range. The increase in composite stiffness with the fiber content suggests a reduction in free volume within the system, causing an improvement in the dimensional stability of the composites, and consequently, an increase in HDT values. Specifically, the incorporation of basalt fibers guaranteed around 75% HDT increment for all of the formulations. These results are particularly interesting because they suggest that these biocomposites have the potential to be applied in applications where the maximum service temperature requirement is below 160 °C.

On the contrary, the addition of short basalt fibers was observed to reduce the Charpy impact strength of polyamide. For the pure polymer, in fact, the impact strength was consistent with the manufacturer’s technical datasheet, which reports a value of 9 kJ/m^2^. Compared to other polyamides, the impact strength of bio-PA11 was higher, as noted by Kawada et al. [77], thereby confirming its suitability for applications where impact resistance is crucial. In any case, the impact strength decreases with the addition of basalt fibers, particularly at the highest fiber concentration of 20% by weight. This reduction is likely to be due to the fiber orientation, which may not be conducive to crack inhibition.

## 4. Conclusions

The sustainability in biobased composite materials is of paramount importance for reducing environmental impact and promoting a circular economy. In this framework, this paper aimed to investigate a novel process pathway to produce compounds and then injection molded items with bio-PA11 as the matrix and different amounts of short basalt fibers. Specifically, the purpose of this work was to understand whether a prediction of fiber breakage in the extrusion process was possible through 1D computation software in order to maximize the macroscopic properties of the final composite.

The results demonstrate that the Ludovic^®^ software provides valuable tools for optimizing the extrusion process and selecting appropriate parameters for the real twin-screw extrusion process. The predictive analysis of fiber breakage indicated that while the model provided a good estimation of the average fiber length, it tended to be overly optimistic regarding the presence of a long tail of a small number of longer fibers that were not present. Additionally, it is noteworthy that the transition from compounding to injection molding did not result in a significant reduction in the average fiber length. Instead, as the fiber content increased, the fiber orientation along the injection flow in the core decreased. The morphological analysis and micro-CT images demonstrated excellent homogenization and distribution of the fibers within the polymer matrix, along with a good degree of adhesion.

Moreover, a comprehensive characterization was performed including morphological and mechanical assessments to evaluate the properties of the produced biocomposite material. The incorporation of 10, 15, and 20 wt.% of basalt fibers into the biobased PA11 resulted in notable enhancements of the material properties. These fibers, known for their exceptional modulus and strength, contributed to improvements in the final performance of the final biocomposites. Notably, the enhancement in heat deflection temperature (HDT) achieved values of 150 °C, along with the improvements in stiffness, flexural properties, and the minimal reductions in impact properties, guaranteeing that these biocomposites could be particularly suitable for automotive interior applications. Future research could focus on surface design and finishing techniques to enhance the esthetic properties. Given that polyamides are prone to moisture absorption, investigating hydrophobic surface coatings could be especially beneficial.

Finally, the adhesion between the fiber and the matrix was found to be robust, as indicated by the correlation between the microscopic structure and the macroscopic mechanical properties and the analytical model employed.

All of these results have led to a wider use of bio-PA11 coupled with chopped basalt fibers. Thanks to the optimization of the extrusion process, short basalt fibers can be used to improve the thermomechanical properties, thereby increasing the range of suitable applications for bio-PA11.

## Figures and Tables

**Table 1:** Biocomposite formulations.

	Bio-Polyamide 11 (wt.%)	Basalt Fibers (wt.%)
**Bio-PA11**	100	0
**Bio-PA11 + 10BF**	90	10
**Bio-PA11 + 15BF**	85	15
**Bio-PA11 + 20BF**	80	20

**Table 2:** Injection molding parameters for the biocomposites with bio-PA11 and different weight percentage of basalt fibers (BFs).

	Bio-PA11	Bio-PA11 + 10BF	Bio-PA11 + 15BF	Bio-PA11 + 20BF
Injection pressure (bar)	70	80	70	70
Holding pressure (bar)	70	80	70	70
Holding time (s)	3	3	3	3
Cooling time (s)	1	1	1	1
Injection speed (%)	70	70	70	70
Temperature profile from the hopper to the mold (°C)	200–220–230	200–220–230	200–220–230	200–220–230
Mold temperature (°C)	25	25	25	25

**Table 3:** Set of input data evaluated for bio-PA11 and basalt fibers and inserted in Ludovic^®^.

***Bio-PA11***	
Melting temperature (°C)	189.3
Melting enthalpy (kJ/kg)	62.8
Solid phase	
Heat capacity (J/kg/°C)	1266.8
Density (kg/m^3^)	1030
Thermal conductivity (W/m·K)	0.23
Liquid phase	
Heat capacity (J/kg/°C)	2072.4
Density (kg/m^3^)	889.5
Thermal conductivity (W/m·K)	0.23
***Basalt fibers***	
Heat capacity (J/kg/°C)	860
Density (kg/m^3^)	2670
Thermal conductivity (W/m/°C)	0.031

**Table 4:** Most promising simulation results showing the parameters selected for the real compounding process.

Screw Speed [RPM]	Flow Rate [kg/h]	Global Mixing Index	T Max (°C)	t in Which T > 230 °C [s]	Viscosity at the Exit [Pa·s]	SME [kWh/t]	RTD [s]
208	11.10	0.39	256	18.00	696.50	176.10	39.21
224	10.20	0.37	241	20.50	627.37	187.70	40.24
224	11.10	0.42	243	18.30	636.04	181.90	38.12
240	10.20	0.41	251	23.00	527.61	194.30	39.28
240	11.10	0.38	260	21.30	581.55	188.10	37.16
256	11.10	0.40	257	22.10	532.46	194.00	36.32

## Data Availability

The original contributions presented in this study are included in the article. Further inquiries can be directed to the corresponding author.

## References

[1] Dace E., Cascavilla A., Bianchi M., Chioatto E., Zecca E., Ladu L., Yilan G. (2024). Barriers to Transitioning to a Circular Bio-Based Economy: Findings from an Industrial Perspective. Sustain. Prod. Consum..

[2] von Vacano B., Mangold H., Vandermeulen G.W.M., Battagliarin G., Hofmann M., Bean J., Künkel A. (2023). Sustainable Design of Structural and Functional Polymers for a Circular Economy. Angew. Chem. Int. Ed..

[3] Sergi C., Tirillò J., Seghini M.C., Sarasini F., Fiore V., Scalici T. (2019). Durability of Basalt/Hemp Hybrid Thermoplastic Composites. Polymers.

[4] Jamshaid H., Mishra R. (2016). A Green Material from Rock: Basalt Fiber—A Review. J. Text. Inst..

[5] Fiore V., Scalici T., Di Bella G., Valenza A. (2015). A Review on Basalt Fibre and Its Composites. Compos. B Eng..

[6] Asadi A., Baaij F., Mainka H., Rademacher M., Thompson J., Kalaitzidou K. (2017). Basalt Fibers as a Sustainable and Cost-Effective Alternative to Glass Fibers in Sheet Molding Compound (SMC). Compos. B Eng..

[7] Fořt J., Kočí J., Černý R. (2021). Environmental Efficiency Aspects of Basalt Fibers Reinforcement in Concrete Mixtures. Energies.

[8] Tavadi A.R., Naik Y., Kumaresan K., Jamadar N.I., Rajaravi C. (2022). Basalt Fiber and Its Composite Manufacturing and Applications: An Overview. Int. J. Eng. Sci. Technol..

[9] Dhand V., Mittal G., Rhee K.Y., Park S.J., Hui D. (2015). A Short Review on Basalt Fiber Reinforced Polymer Composites. Compos. B Eng..

[10] Gao Y.Q., Jia C., Meng L., Li X.H. (2017). Heat Resistance Study of Basalt Fiber Material via Mechanical Tests. IOP Conf. Ser. Mater. Sci. Eng..

[11] Khandelwal S., Rhee K.Y. (2020). Recent Advances in Basalt-Fiber-Reinforced Composites: Tailoring the Fiber-Matrix Interface. Compos. B Eng..

[12] Bednarowski D., Bazan P., Kuciel S. (2023). Enhancing Strength and Sustainability: Evaluating Glass and Basalt Fiber-Reinforced Biopolyamide as Alternatives for Petroleum-Based Polyamide Composite. Polymers.

[13] Aslam Shaikh A., Anil Pradhan A., Mahesh Kotasthane A., Patil S., Karuppanan S. (2022). Comparative Analysis of Basalt/E-Glass/S2-Fibreglass-Carbon Fiber Reinforced Epoxy Laminates Using Finite Element Method. Mater. Today Proc..

[14] Sergi C., Botta L., Tirillò J., Sarasini F. (2024). Basalt/Polypropylene Composites: The Effects of Mechanical Reprocessing on Their Morphological, Thermal, Rheological and Mechanical Behavior. Mater. Today Sustain..

[15] Gurunathan T., Mohanty S., Nayak S.K. (2015). A Review of the Recent Developments in Biocomposites Based on Natural Fibres and Their Application Perspectives. Compos. Part A Appl. Sci. Manuf..

[16] Khan F., Hossain N., Hasan F., Rahman S.M., Khan S., Safiullah A.Z.A., Chowdhury M.A. (2024). Advances of natural fiber composites in diverse engineering applications—A review. Appl. Eng. Sci..

[17] Sergi C., Vitiello L., Russo P., Tirillò J., Sarasini F. (2022). Toughened Bio-Polyamide 11 for Impact-Resistant Intraply Basalt/Flax Hybrid Composites. Macromol.

[18] Jariyavidyanont K., Focke W., Androsch R., Di Lorenzo M.L., Androsch R. (2019). Thermal Properties of Biobased Polyamide 11. Thermal Properties of Bio-Based Polymers.

[19] Khedr M.S.F. (2023). Bio-Based Polyamide. Phys. Sci. Rev..

[20] Di Lorenzo M.L., Longo A., Androsch R. (2019). Polyamide 11/Poly(Butylene Succinate) Bio-Based Polymer Blends. Materials.

[21] Yoshida T., Touji M., Takagi H., Shimizu N., Igarashi N., Sakurai S., Uchida M., Kaneko Y. (2024). Structure and Mechanical Properties of Biobased Polyamide 11 Specimens Subjected to Different Heat Treatments. Polym. J..

[22] Jariyavidyanont K., Williams J.L., Rhoades A.M., Kühnert I., Focke W., Androsch R. (2018). Crystallization of Polyamide 11 during Injection Molding. Polym. Eng. Sci..

[23] Prasad V., Alliyankal Vijayakumar A., Jose T., George S.C. (2024). A Comprehensive Review of Sustainability in Natural-Fiber-Reinforced Polymers. Sustainability.

[24] Greco A., Maffezzoli A., Casciaro G., Caretto F. (2014). Mechanical Properties of Basalt Fibers and Their Adhesion to Polypropylene Matrices. Compos. B Eng..

[25] Raajeshkrishna C.R., Pradeep A.S., Rishi Kumar R.D. (2019). Influence of Fiber Content on Mechanical, Tribological Properties of Short Basalt Fiber-Reinforced Nylon 6 and Polypropylene Composites. J. Thermoplast. Compos. Mater..

[26] Blackman Z., Olonisakin K., MacFarlane H., Rodriguez-Uribe A., Tripathi N., Mohanty A.K., Misra M. (2024). Sustainable Basalt Fiber Reinforced Polyamide 6,6 Composites: Effects of Fiber Length and Fiber Content on Mechanical Performance. Compos. Part. C Open Access.

[27] Vitiello L., Russo P., Papa I., Lopresto V., Mocerino D., Filippone G. (2021). Flexural Properties and Low-Velocity Impact Behavior of Polyamide 11/Basalt Fiber Fabric Laminates. Polymers.

[28] Papa I., Bruno M., Napolitano F., Esposito L., Lopresto V., Russo P. (2024). Numerical and Mechanical Analysis of Laminated PA11/Twill Basalt Composites with Enhanced Flame Behavior. Compos. Struct..

[29] Barczewski M., Hejna A., Andrzejewski J., Aniśko J., Piasecki A., Mróz A., Ortega Z., Rutkowska D., Sałasińska K. (2024). The Recyclability of Fire-Retarded Biobased Polyamide 11 (PA11) Composites Reinforced with Basalt Fibers (BFs): The Influence of Reprocessing on Structure, Properties, and Fire Behavior. Molecules.

[30] Coughlin N., Drake B., Fjerstad M., Schuster E., Waege T., Weerakkody A., Letcher T. (2019). Development and Mechanical Properties of Basalt Fiber-Reinforced Acrylonitrile Butadiene Styrene for In-Space Manufacturing Applications. J. Compos. Sci..

[31] Sam-Daliri O., Ghabezi P., Steinbach J., Flanagan T., Finnegan W., Mitchell S., Harrison N. (2023). Experimental Study on Mechanical Properties of Material Extrusion Additive Manufactured Parts from Recycled Glass Fibre-Reinforced Polypropylene Composite. Compos. Sci. Technol..

[32] Ning F., Cong W., Qiu J., Wei J., Wang S. (2015). Additive Manufacturing of Carbon Fiber Reinforced Thermoplastic Composites Using Fused Deposition Modeling. Compos. B Eng..

[33] Zhuang H., Pu R., Zong Y., Dai G.C. (2008). Relationship between Fiber Degradation and Residence Time Distribution in the Processing of Long Fiber Reinforced Thermoplastics. Express Polym. Lett..

[34] Deák T., Czigány T., Maršálková M., Militký J. (2010). Manufacturing and Testing of Long Basalt Fiber Reinforced Thermoplastic Matrix Composites. Polym. Eng. Sci..

[35] Adole O., Anguilano L., Minton T., Campbell J., Sean L., Valisios S., Tarverdi K. (2020). Basalt Fibre-Reinforced High Density Polyethylene Composite Development Using the Twin Screw Extrusion Process. Polym. Test..

[36] Aliotta L., Gasenge M., Gigante V., Lazzeri A. (2022). Micromechanical Deformation Processes and Failure of PBS Based Composites Containing Ultra-Short Cellulosic Fibers for Injection Molding Applications. Polymers.

[37] Durin A., De Micheli P., Nguyen H.-C., David C., Valette R., Vergnes B. (2014). Comparison between 1D and 3D Approaches for Twin-Screw Extrusion Simulation. Int. Polym. Process..

[38] Gigante V., Aliotta L., Dal Pont B., Titone V., Botta L., La Mantia F.P., Lazzeri A. (2023). Tailoring Morphology and Mechanical Properties of PLA/PBSA Blends Optimizing the Twin-Screw Extrusion Processing Parameters Aided by a 1D Simulation Software. Polym. Test..

[39] Gigante V., Gallone G., Aliotta L., Lazzeri A. (2024). Twin-Screw Extrusion Optimization and Study of Morphological, Thermal, Mechanical and Fracture Properties of Sustainable Poly(Lactic Acid) (PLA) and Poly(Butylene Sebacate) (PBSe) Blends. Mater. Today Sustain..

[40] Aliotta L., Lazzeri A. (2020). A Proposal to Modify the Kelly-Tyson Equation to Calculate the Interfacial Shear Strength (IFSS) of Composites with Low Aspect Ratio Fibers. Compos. Sci. Technol..

[41] Pearson A., Liao W., Kazemi Y., Duncan M., Slingerland E., Kakroodi A., Heydrich M., Hammami A., Naguib H.E. (2022). Fiber-Matrix Adhesion between High-Density Polyethylene and Carbon Fiber. Polym. Test..

[42] (2022). Standard Test Methods for Determining the Biobased Content of Solid, Liquid, and Gaseous Samples Using Radiocarbon Analysis.

[43] Hassan A., Yahya R., Rafiq M.I.M., Hussin A., Sheikh M.R.K., Hornsby P.R. (2011). Interfacial Shear Strength and Tensile Properties of Injection-Molded, Short- and Long-Glass Fiber-Reinforced Polyamide 6,6 Composites. J. Reinf. Plast. Compos..

[44] (2019). Plastics—Determination of tensile properties.

[45] Asoodeh F., Aghvami-Panah M., Salimian S., Naeimirad M., Khoshnevis H., Zadhoush A. (2022). The Effect of Fibers’ Length Distribution and Concentration on Rheological and Mechanical Properties of Glass Fiber–Reinforced Polypropylene Composite. J. Ind. Text..

[46] (2017). Standard Test Method for Tensile Properties of Polymer Matrix Composite Materials.

[47] (2021). Standard Test Methods for Flexural Properties of Unreinforced and Reinforced Plastics and Electrical Insulating Materials.

[48] (2023). Plastics—Determination of Charpy Impact Properties—Part 1: Non-Instrumented Impact Test.

[49] Papanicolaou G.C., Portan D.V., Kontaxis L.C. (2021). Interrelation between Fiber–Matrix Interphasial Phenomena and Flexural Stress Relaxation Behavior of a Glass Fiber–Polymer Composite. Polymers.

[50] Jiang Q., Takayama T., Nishioka A. (2024). Improving the Accuracy of the Evaluation Method for the Interfacial Shear Strength of Fiber-Reinforced Thermoplastic Polymers through the Short Beam Shear Test. Polymers.

[51] Mani A., Sharma S. (2022). Interfacial Shear Strength of Carbon Nanotube Reinforced Polymer Composites: A Review. Mater. Today Proc..

[52] Topçu M., Madabhushi G.S.P., Staat M. (2022). A Generalized Shear-Lag Theory for Elastic Stress Transfer between Matrix and Fibres Having a Variable Radius. Int. J. Solids Struct..

[53] Kelly A., Tyson W.R. (1965). Tensile Properties of Fibre-Reinforced Metals: Copper/Tungsten and Copper/Molybdenum. J. Mech. Phys. Solids.

[54] Perolo A., Castiglioni A., Ferri D. (2017). Electrification of Polymers during Capillary Extrusion. AIP Conf. Proc..

[55] Yang Y., Mark J.E. (2007). Thermal Conductivity. Physical Properties of Polymers Handbook.

[56] Grzesiak S., Pahn M., Klingler A., Akpan E.I., Schultz-Cornelius M., Wetzel B. (2022). Mechanical and Thermal Properties of Basalt Fibre Reinforced Polymer Lamellas for Renovation of Concrete Structures. Polymers.

[57] D’Anna A., Arrigo R., Frache A. (2022). Rheology, Morphology and Thermal Properties of a PLA/PHB/Clay Blend Nanocomposite: The Influence of Process Parameters. J. Polym. Environ..

[58] Hyvärinen M., Jabeen R., Kärki T. (2020). The Modelling of Extrusion Processes for Polymers—A Review. Polymers.

[59] del Pilar Noriega E.M., Rauwendaal C., del Pilar Noriega E.M., Rauwendaal C. (2019). Troubleshooting the Extrusion Process. Troubleshooting the Extrusion Process.

[60] Aho J. (2011). Rheological Characterization of Polymer Melts in Shear and Extension: Measurement Reliability and Data for Practical Processing.

[61] Consul P., Beuerlein K.-U., Luzha G., Drechsler K. (2021). Effect of Extrusion Parameters on Short Fiber Alignment in Fused Filament Fabrication. Polymers.

[62] de Lima T., de Azevedo A., Marvila M., Candido V., Fediuk R., Monteiro S. (2022). Potential of Using Amazon Natural Fibers to Reinforce Cementitious Composites: A Review. Polymers.

[63] Huang S., Fu Q., Yan L., Kasal B. (2021). Characterization of Interfacial Properties between Fibre and Polymer Matrix in Composite Materials—A Critical Review. J. Mater. Res. Technol..

[64] Closse A., Onesippe Potiron C., Arsene M.-A., Bilba K. (2024). Assessment of Vegetable Fiber-Matrix Adhesion and Durability, in Cement-Based and Polymer-Based Composite: Principles from Literature Review. J. Compos. Mater..

[65] Lee C.H., Khalina A., Lee S.H. (2021). Importance of Interfacial Adhesion Condition on Characterization of Plant-Fiber-Reinforced Polymer Composites: A Review. Polymers.

[66] Tseng H.-C., Chang R.-Y., Hsu C.-H. (2017). Improved Fiber Orientation Predictions for Injection Molded Fiber Composites. Compos. Part. A Appl. Sci. Manuf..

[67] Ralph C., Lemoine P., Archer E., McIlhagger A. (2019). Mechanical Properties of Short Basalt Fibre Reinforced Polypropylene and the Effect of Fibre Sizing on Adhesion. Compos. B Eng..

[68] Bowyer W.H., Bader M.G. (1972). On the Re-Inforcement of Thermoplastics by Imperfectly Aligned Discontinuous Fibres. J. Mater. Sci..

[69] Thomason J.L. (2005). The Influence of Fibre Length and Concentration on the Properties of Glass Fibre Reinforced Polypropylene. 6. The Properties of Injection Moulded Long Fibre PP at High Fibre Content. Compos. Part A Appl. Sci. Manuf..

[70] Fu Q., Xu W., Bu M., Guo B., Niu D. (2021). Effect and Action Mechanism of Fibers on Mechanical Behavior of Hybrid Basalt-Polypropylene Fiber-Reinforced Concrete. Structures.

[71] Jagadeesh P., Rangappa S.M., Siengchin S. (2024). Basalt Fibers: An Environmentally Acceptable and Sustainable Green Material for Polymer Composites. Constr. Build. Mater..

[72] Venkateshwaran N., ElayaPerumal A., Jagatheeshwaran M.S. (2011). Effect of Fiber Length and Fiber Content on Mechanical Properties of Banana Fiber/Epoxy Composite. J. Reinf. Plast. Compos..

[73] Aliotta L., Gigante V., Coltelli M.B., Cinelli P., Lazzeri A. (2019). Evaluation of Mechanical and Interfacial Properties of Bio-Composites Based on Poly(Lactic Acid) with Natural Cellulose Fibers. Int. J. Mol. Sci..

[74] Song K., Yu Y., Liu Y., Zhao J. (2023). Flexural Performance Study of Basalt-Fiber-Reinforced Polymer Bar Basalt-Fiber-Reinforced Concrete Beams. Buildings.

[75] Li G., Zhang S., Yu H., Liu G., Wang W., Chen G., Wang J., Wan W., Lu Q., Chen H. (2024). Enhancing the Mechanical Properties of Basalt Fiber/Nylon 6 Composites by Surface Roughening and Hydrogen Bonding Interaction. Polym. Compos..

[76] Panthapulakkal S., Sain M. (2007). Injection-molded Short Hemp Fiber/Glass Fiber-reinforced Polypropylene Hybrid Composites—Mechanical, Water Absorption and Thermal Properties. J. Appl. Polym. Sci..

[77] Kawada J., Kitou M., Mouri M., Mitsuoka T., Araki T., Lee C.-H., Ario T., Kitou O., Usuki A. (2016). Morphology Controlled PA11 Bio-Alloys with Excellent Impact Strength. ACS Sustain. Chem. Eng..

